# First Survey of Metallo-β–Lactamase Producers in Clinical Isolates of *Pseudomonas aeruginosa* From a Referral Burn Center in Kurdistan Province

**DOI:** 10.17795/jjnpp-3546

**Published:** 2012-01-04

**Authors:** Enayatollah Kalantar, Vahideh Torabi, Heiman Salimizand, Fariborz Soheili, Soheila Beiranvand, Mohammad Mehdi Soltan Dallal

**Affiliations:** 1 Envirronmental Health Resaerch Center, Kurdistan University of Medical Sciences, Sanandaj, IR Iran; 2 Department of Biological Sciences, Islamic Azad University, Sciences Research Branch, Tehran, IR Iran; 3 Department of Microbiology, School of Medicine, Kurdistan University of Medical Sciences, Sanandaj, IR Iran; 4 School of Medicine, Kurdistan University of Medical Sciences, Sanandaj, IR Iran; 5 Qods Hospital, Kurdistan University of Medical Sciences, Sanandaj, IR Iran; 6 Division of Microbiology, School of Public Health, Tehran University of Medical Sciences, Tehran, IR Iran

**Keywords:** Burn Patients, *P. aeruginosa*, Metallo-β-lactamase, Imipenem Resistance

## Abstract

**Background:**

Treatment of infectious diseases is becoming more challenging with each passing year. This is especially true for infections caused by Pseudomonas aeruginosa, an opportunistic pathogen with the ability to rapidly develop resistance to multiple classes of antibiotics.

**Objectives:**

This study was conducted to determine the prevalence of metallo-β-lactamase (MBL)–producing strains among multidrug-resistant P. aeruginosa strains isolated from burn patients.

**Materials and Methods:**

The isolates were identified, tested for susceptibility to various antimicrobial agents, and screened for the presence of MβLs by using the double-disk synergy test. The minimal inhibitory concentration of imipenem was determined by microplate broth dilution method on Mueller-Hinton agar. To detect VIM, SIM, and GIM MBLs, the isolates were subjected to polymerase chain reaction.

**Results:**

In this study, we identified 100 P. aeruginosa isolates from 176 clinical specimens obtained from burn patients. The isolates showed maximum resistance to ampicillin (100%), ceftazidime (94%), and ceftriaxone (89%). The CLSI-MBL phenotypic test showed that of the 100 P. aeruginosa isolates, 22 (22%) were positive for MBL production in the double-disk synergy test. Of the 22 MBL-positive P. aeruginosa isolates, 8 were resistant to imipenem. PCR analysis showed that 8 isolates were positive for blaVIM1. The other genes blaSIM1 and blaGIM1 were not detected.

**Conclusions:**

The study results demonstrate the serious therapeutic threat of the spread of MBL producers among P. aeruginosa populations. Metallo-β-lactamases were detected in 22% of imipenem-resistant P. aeruginosa isolates. Early detection and infection-control practices are the best antimicrobial strategies for this organism; therefore, systematic surveillance to detect MBL producers is necessary.

## 1. Background

*Pseudomonas aeruginosa*, a versatile pathogen associated with a broad spectrum of infections in humans and widely known as an opportunistic organism, is frequently involved in infections among susceptible populations, especially patients with burns (1). Furthermore, this organism possesses several virulence factors and is intrinsically resistant to most antimicrobials, a feature that is also responsible for the difficulty in treating infected patients (2). In the past decade, *P. aeruginosa* strains showing resistance to multiple β-lactam antibiotics, in particular, metallo-β-lactamase (MBL)–producing *P. aeruginosa*, have become an increasing public health problem worldwide (3-5). Furthermore, infection with MBL-producing organisms is associated with higher rates of mortality, morbidity, and health care costs (6-8).

In *P. aeruginosa*, resistance to extended-spectrum β-lactams is mediated by lack of drug penetration, which may occur due to porin mutations, efflux pumps, or hydrolysis by β-lactamases (9). On the basis of molecular studies, we can classify carbapenem-hydrolyzing enzymes into 4 groups: A, B, C, and D. The MBLs, which belong to group B, are enzymes that require divalent cations as cofactors for optimal enzyme activity and are inhibited by the action of a metal ion chelator. Hospital infections caused by *P. aeruginosa* are often difficult to eradicate because the organisms are resistant to drugs. Therefore, detection of MBL-producing *P. aeruginosa* is crucial to control the spread of resistance and for optimal treatment of patients, especially burn patients (10). There is no information concerning the prevalence of MBL-producing *P. aeruginosa* strains in Kurdistan province. 

## 2. Objectives

We conducted this study to detect MBL-producing strains among *P. aeruginosa* isolates obtained from burn patients at Tohid hospital, which is affiliated to Kurdistan University of Medical Sciences and has a burn unit with a heavy patient turnover and extensive antibiotic use.

## 3. Materials and Methods

Between April 2009 and April 2010, we isolated and identified 100 strains of *P. aeruginosa* in a clinical laboratory at Tohid hospital, Sanandaj, Iran. The strains were identified as *P. aeruginosa* on the basis of colony morphology, gram staining results, motility, oxidase reaction, production of the pyocyanin pigment, nitrate reduction, the use of citrate and malonate as carbon sources, and the ability to grow at 5˚C and 42˚C.

### 3-1. Antimicrobial Susceptibility Testing 

Antimicrobial susceptibility testing was done by the disk-diffusion method on Muller-Hinton agar (Merck, Germany) (11). The following antibiotics were used: amikacin, gentamicin, carbencillin, ciprofloxacin, ofloxacin, cefepime, ceftazidime, cefotaxime, ampicillin, ceftriaxone, imipenem, and meropenem.

### 3-2. Detection of MBL 

Detection of MBL-producing *P. aeruginosa* strains was performed according to Clinical and Laboratory Standards Institute (CLSI) guidelines (11). *P. aeruginosa* ATCC 27853 was used as the negative control. For the double-disk synergy test, the inoculum was prepared after emulsifying 5–6 colonies of the suspected isolate in Mueller–Hinton broth, and the turbidity was adjusted to 0.5 McFarland opacity standard. A lawn culture was obtained on Mueller–Hinton agar, and double-disk synergy test was performed. 

### 3-3. Determination of Minimal Inhibitory Concentration 

Imipenem minimal inhibitory concentration (MIC) was determined by microplate (NUNC, Denmark) broth dilution method on Mueller–Hinton agar. Zone diameter and MIC interpretive standards for breakpoints for *P. aeruginosa* published by CLSI were used (11). 

### 3-4 Genotypic Detection of VIM, SIM, and GIM MBL Genes by PCR

To detect VIM, SIM, and GIM MBL genes, PCR was performed for *P. aeruginosa* isolates (12) 

## 4. Results

**Figure 1:**
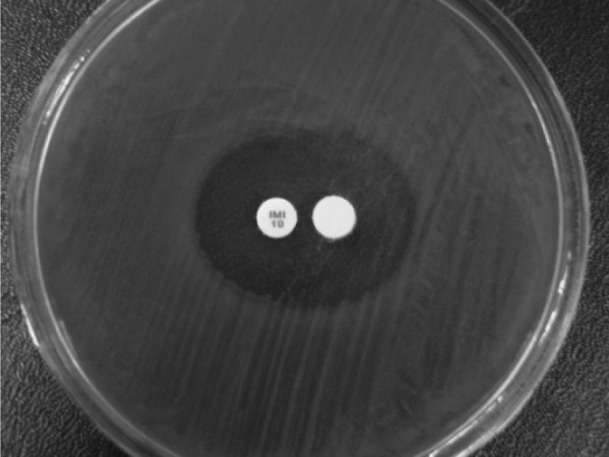
Phenotypic Detection of MBLs by DDST Among P. aeruginosa Isolates in Burn Patients at Tohid Hospital, Sanandaj, Iran

In this study, we identified 100 *P. aeruginosa* isolates among 176 clinical specimens obtained from patients. Table 1 shows the antibiotic-resistance pattern of *P. aeruginosa* isolates. The isolates showed maximum resistance to ampicillin (100%), ceftazidime (94%), and ceftriaxone (89%). The next step in testing was designed to identify the MBL-producing *P. aeruginosa* strains. In the CLSI-MBL phenotypic test, of the 100 *P. aeruginosa* isolates, 22 (22%) were positive for MBL production by the double-disk synergy test (Figure 1).

**Table 1:** In Vitro Antibiotic Resistance Pattern of Infection-Associated P. aeruginosa

Antibiotic	Resistance, %
Amikacin	52
Gentamicin	54
Carbencillin	76
Ciprofloxacin	31
Ofloxacin	82
Cefepime	72
Ceftazidime	96
Cefotaxime	88
Ampicillin	100
ceftriaxone	89
Imipenem	21
Meropenem	14

### 4-1 Determination of MIC

**Figure 2:**
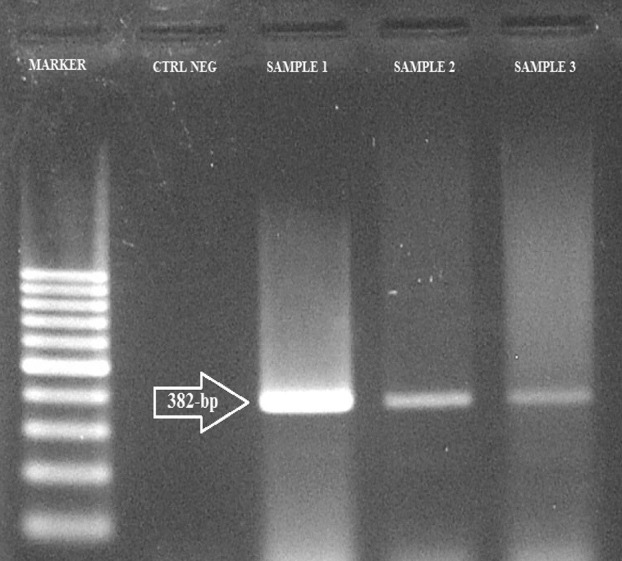
PCR for the Detection of blaVIM Gene

The MIC of MBL-positive isolates for imipenem is shown in Table 2; among 22 MBL-positive strains, 8 were resistant. PCR detection for MBL showed that 8 strains were positive for bla_VIM1_. The other genes, bla_SIM1_ and bla_GIM1_, were not detected (Figure 2).

**Table 2:** MIC of MBL positive isolates for Imipenem

Sensitive	Intermediate	Resistant
	MIC^a^, μg / ml	
≤ 0.125	08	16
0.25		32
0.5		64
01		128 ≤
02		
04		
	No. of *P. aeruginosa* Isolates, %	
02	01	02
02		01
04		03
02		02
03		
00		
	Total of *P. aeruginosa* Isolates	
13	01	08

^a^Abbreviation: MIC: Minimum Inhitory Concentration

## 5. Discussion

Because of its broad antimicrobial spectrum and stability against most common β-lactamases, imipenem generally represents one of the last alternatives for the treatment of nosocomial infections caused by multidrug-resistant gram-negative bacteria, particularly *P. aeruginosa* (13). However, the rapid spread of MBLs among major gram-negative pathogens, particularly *P. aeruginosa*, is an emerging threat and a matter of concern worldwide (14-16); further, MBL-carrying bacteria are known to cause recalcitrant nosocomial infections (17). MBLs of the IMP, VIM, and SIM families are frequently detected in imipenem-resistant *P. aeruginosa* in Iran (18-20). In our study, a total of 100 *P. aeruginosa* strains were isolated from hospitalized burn patients in Sanandaj, Iran, in 2010, and 22% of these strains were found to be MBL producers, which is much lower than the findings from the study conducted by Mihani and Khosravi in Ahvaz (18). In this study, 22% of the imipenem-resistant *P. aeruginosa* isolates were MBL-positive, with 8% positive for VIM1, which is by far the most prevalent MBL in Iran^20^. To our knowledge, MBLs of other families like SIM have not been detected in *P. aeruginosa*. The prevalence of MBL-producing *P. aeruginosa* differs across Iranian studies, which may be because of differences in geographic regions. Further investigation is required to gain a better understanding of the epidemiology and genetic background of MBL-producing *P. aeruginosa*. The uncontrolled spread of MBL producers in hospitals may hamper treatment procedures and lead to increased morbidity and mortality. Systems for regular screening should be established to control the spread of MBL producers, and effective infection-control programs in hospitals should be developed and implemented thoroughly. The study results demonstrate the serious therapeutic threat of the spread of MBL-producing *P. aeruginosa*. This number (22% of imipenem-resistant *P. aeruginosa*) might have been higher if other genes were included. Early detection and infection control practices are the best defense against this organism; therefore, systematic surveillance to detect MBL producers is necessary. 
